# N-Acetyl-Aspartate in the dorsolateral prefrontal cortex in men with schizophrenia and auditory verbal hallucinations: A 1.5 T Magnetic Resonance Spectroscopy Study

**DOI:** 10.1038/s41598-018-22597-y

**Published:** 2018-03-07

**Authors:** Marion Psomiades, Marine Mondino, Clara Fonteneau, Remy Bation, Frederic Haesebaert, Marie-Françoise Suaud-Chagny, Jerome Brunelin

**Affiliations:** 1 INSERM, U1028; CNRS, UMR5292; Lyon Neuroscience Research Center, Psychiatric Disorders: from Resistance to Response Team, Lyon, F-69000 France; 2 University Lyon 1, Villeurbanne, F-69000 France; 3 Centre Hospitalier Le Vinatier, Bron, France

## Abstract

Auditory verbal hallucinations (AVH) in patients with schizophrenia are linked to abnormalities within a large cerebral network including frontal and temporal regions. Whilst abnormalities of frontal speech production and temporal speech perception regions have been extensively studied, alterations of the dorsolateral prefrontal cortex (DLPFC), a region critically involved in the pathophysiology of schizophrenia, have rarely been studied in relation to AVH. Using 1.5 T proton magnetic resonance spectroscopy, this study examined the relationship between right and left DLPFCs N-AcetylAspartate (NAA) levels and the severity of AVH in patients with schizophrenia. Twenty-seven male patients with schizophrenia were enrolled in this study, 15 presented daily treatment-resistant AVH (AVH+) and 12 reported no AVH (no-AVH). AVH+ patients displayed higher NAA levels in the right DLPFC than no-AVH patients (p = 0.033). In AVH+ patients, NAA levels were higher in the right DLPFC than in the left (p = 0.024). No difference between the right and left DLPFC was observed in no-AVH patients. There was a positive correlation between NAA levels in the right DLPFC and the severity of AVH (r = 0.404, p = 0.037). Despite limited by magnetic field strength, these results suggest that AVH may be associated with increased NAA levels in the right DLPFC in schizophrenia.

## Introduction

Auditory verbal hallucinations (AVH) are a disabling and frequent symptom in patients with schizophrenia. Classically, AVH have been linked to abnormal activities within bilateral inferior frontal and temporal regions associated with speech generation and speech perception^1,2^. Also, patients with AVH have been reported to display abnormal structural^3,4^ and functional^5^ fronto-temporal connectivity and abnormal inter-hemispheric connections between auditory cortices^6^. However, language-related areas are not the only brain structures involved in the generation of AVH. Numerous functional magnetic resonance imaging (fMRI) studies highlighted the involvement of the left^5,7–10^ and the right^1,7,8,11^ dorsolateral prefrontal cortex (DLPFC) in AVH. However, only few studies have explored the functional connectivity of the right DLPFC by comparing patients with AVH (AVH+patients) and patients with no AVH (no-AVH patients). In this line, measuring local neuronal activity by regional homogeneity analysis of BOLD, Cui *et al*.^8^ put forward an increased contribution of the right DLPFC in AVH+ patients compared to no-AVH patients. At the neurochemical level, only few studies have investigated the contribution of the DLPFC in AVH in patients with schizophrenia. In a recent study using proton magnetic resonance spectroscopy (^1^H-MRS), Ćurčić-Blake *et al*.^12^ reported higher Glx levels in the left DLPFC in AVH+ patients compared with no-AVH patients whereas the right part of the DLPFC was not considered. When measured by *in vivo* ^1^H-MRS, Glx is an index of glutamate and glutamine levels. This result suggested a relationship between high glutamatergic neurotransmission in the left DLPFC and AVH in schizophrenia. Another important brain metabolite that is often investigated using ^1^H-MRS is N-AcetylAspartate (NAA). NAA is the second most abundant amino acid in the brain after glutamate and is exclusively synthetized in neuronal mitochondria. It is described as a reliable marker of neuronal viability and functioning^13,14^. Evidence suggests that NAA levels are decreased in the DLPFC of patients with schizophrenia^15^. However, more studies are needed to directly investigate the relationship between NAA levels in each hemispheric part of the DLPFC and AVH in schizophrenia.

Here, our objective was to study the link between AVH and the DLPFC viability and function as measured by NAA levels. We proposed to compare NAA levels in the left and right DLPFC of AVH+ and no-AVH patients. Based on Cui *et al*.^8^ showing an increased contribution of the right DLPFC in AVH+ patients as compared with no-AVH patients, we hypothesized that AVH+ patients would display higher NAA levels in the right DLPFC as compared to no-AVH patients.

Moreover, as an exploratory outcome, we conducted correlation analyses to investigate the relationship between AVH severity and left and right DLPFC NAA levels.

## Results

### Sociodemographic and clinical characteristics of patients

Twenty-seven male patients with schizophrenia presenting with treatment-resistant symptoms were recruited in this study. The sample was separated into two groups based on the presence and the absence of daily AVH (AVH+ and no-AVH patients, respectively). The final analyzed samples consisted of 15 AVH+ patients with daily AVH (Positive and Negative Syndrome Scale (PANSS) P3 item > 3), and 12 no-AVH patients with no current or past history of AVH (PANSS P3 item ≤ 3). There was no difference between groups regarding age, medication, duration and severity of the illness (PANSS score). The characteristics of patients are given in Table 1.

**Table 1:** Sociodemographic and clinical characteristics of patients with schizophrenia with (AVH+) and without (no-AVH) auditory verbal hallucinations (AVH).

	AVH+	no-AVH	p
n	15	12	
Age (years)	33.9 (4.9)	37.3 (7.3)	0.16
Educational level	10.8 (2.6)	11 (2.7)	0.83
Illness duration (years)	10.8 (5.2)	13.5 (7.3)	0.27
Medication (eq cpz mg/day)	759.5 (483.8)	546.6 (641.8)	0.36
PANSS total	73.9 (13)	75.3 (11.9)	0.77
P3 item (AVH)	5.7 (0.7)	1.5 (0.9)	<0.001

The results are given as the mean (standard deviation).

PANSS: Positive And Negative Syndrome Scale. eq cpz: chlorpromazine equivalent.

### Levels of NAA in the left and right DLPFC in AVH+ and no-AVH male patients

**Figure 1:**
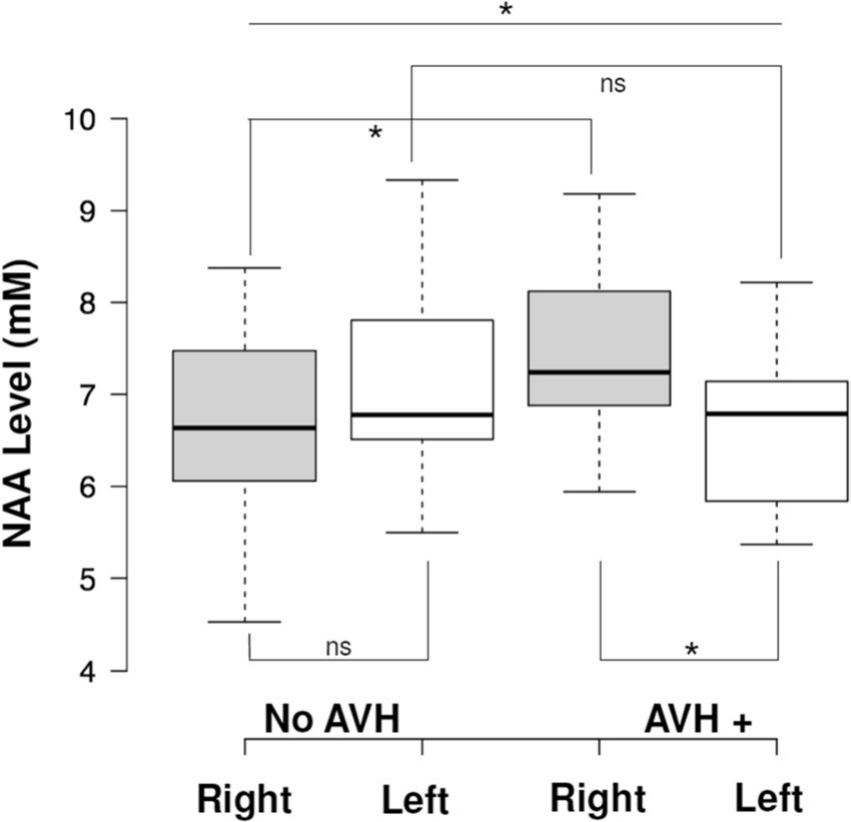
N-AcetylAspartate (NAA) levels measured by ^1^H Magnetic Resonance Spectroscopy in the left and right Dorsolateral Prefrontal Cortex (DLPFC) in patients with schizophrenia with (AVH+, n = 15) and without (no-AVH, n = 12) auditory verbal hallucinations (AVH). NAA levels were higher in the right DLPFC in AVH+ patients than in non-AVH patients and in the left DLPFC (F_(1,25)_ = 5.808; partial η² = 0.189; p = 0.024). Center lines show the medians; box limits indicate the 25th and 75th percentiles; whiskers extend 1.5 times the interquartile range from the 25th and 75th percentiles.

A mixed model ANOVA with group (AVH+, no-AVH) as between factor and laterality (left DLPFC, right DLPFC) as within factor revealed a significant interaction between group and laterality (F_(1,25)_ = 5.808; partial η² = 0.189; p = 0.024; Fig. 1). No main effects of laterality (F_(1,25)_ = 0.644; partial η² = 0.025; p = 0.43) or group (F_(1,25)_ = 0.552; partial η² < 0.022; p = 0.47) were reported.

Post hoc analyses revealed a significant difference between groups for NAA levels in the right DLPFC (p = 0.033). NAA levels in the right DLPFC were higher in AVH+ patients (mean = 7.54 ± standard deviation = 0.97) than in no-AVH patients (6.67 ± 1.02). No difference between groups was reported for NAA levels in the left DLPFC (p = 0.219).

In AVH+ patients, NAA levels were significantly higher in the right DLPFC (7.54 ± 0.97) than in the left DLPFC (6.63 ± 0.91; p = 0.024). In no-AVH patients, there was no difference between NAA levels in the left DLPFC (7.12 ± 1.08) and in the right DLPFC (6.67 ± 1.02; p = 0.29).

### Relationship between NAA levels and severity of AVH

**Figure 2:**
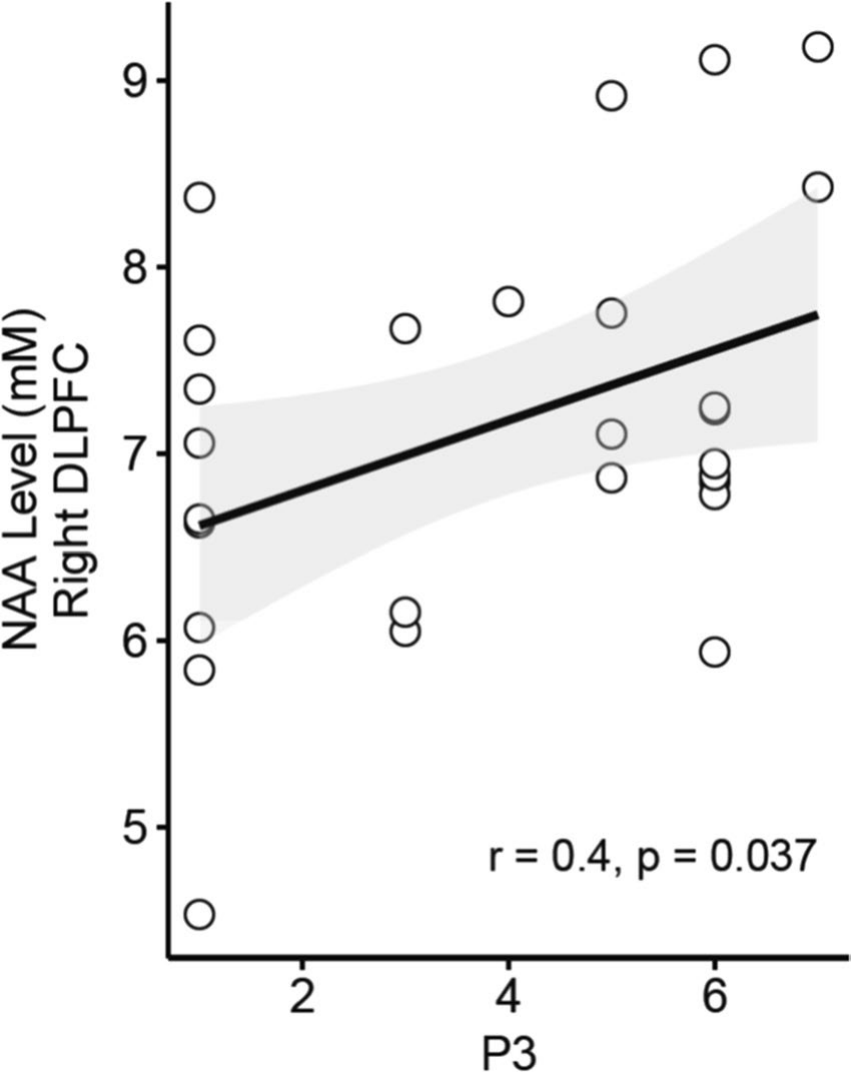
Relationship between N-AcetylAspartate (NAA) levels in the right Dorsolateral Prefrontal Cortex (DLPFC) and severity of auditory verbal hallucinations (AVH) measured by Positive And Negative Syndrome Scale - PANSS P3 item in patients with schizophrenia. (r = 0.404; p = 0.037).

A significant correlation was observed between NAA levels in the right DLPFC and the severity of AVH measured by P3 PANSS item (r = 0.404; p = 0.037; Fig. 2).

No significant correlation was reported between NAA levels in the left DLPFC and AVH severity (r = −0.325; p = 0.098).

## Discussion

In this study we used ^1^H MRS to assess NAA levels in the left and right DLPFC in AVH+ and no-AVH male patients with schizophrenia. We reported that NAA levels were higher in the right DLPFC as compared with the left DLPFC in AVH+ patients. No difference in NAA levels was observed between left and right DLPFC in no-AVH patients. NAA levels in the right DLPFC were higher in AVH+ than in no-AVH patients and no between-group differences were reported in the left DLPFC. To the best of our knowledge, this study is the first to report a significant difference in NAA levels measured in the right DLPFC between two sub-groups of patients with schizophrenia based on the presence of AVH. Indeed, in the literature, the majority of studies that investigated prefrontal NAA levels in schizophrenia has compared patients with schizophrenia with healthy subjects and has measured NAA levels within the DLPFC regardless of the laterality or only in the left DLPFC. These studies showed conflicting results: some reported a decrease in NAA levels measured in the left DLPFC in patients with schizophrenia compared to healthy subjects and some failed to find significant differences^15^. These discrepancies could be explained by the clinical heterogeneity of patients’ symptomatology. Indeed, only two studies have investigated prefrontal NAA levels in relation with schizophrenia symptomatology. The first study by Callicott *et al*.^16^ reported a negative correlation between the NAA/Creatine levels in the DLPFCs and the severity of negative symptoms. However, the authors have not investigated separately the relationship between the left and the right DLPFC and the severity of symptoms and used a mean ratio of NAA/Creatine in the both DLPFCs. The second study by Sigmundsson *et al*.^17^ found a negative correlation between the severity of positive symptoms and NAA levels in the right DLPFC but not between the severity of positive symptoms and NAA levels in the left DLPFC. Moreover, the severity of general symptoms negatively correlated with NAA levels in the left and in the right DLPFC. However, these studies did not permit to isolate DLPFC hemispheric abnormalities linked to AVH. This is one of the reason we chose to directly compare AVH+ to no-AVH-patients.

The current findings of increased NAA levels in the right DLPFC in AVH+ patients as compared to no-AVH patients are consistent with some recent neuroimaging studies showing an increased contribution of right DLPFC in AVH+ patients^1,8,11^. For instance, Cui *et al*.^8^ recently reported higher regional brain activation in the right DLPFC in AVH+ patients as compared with no-AVH patients. Using resting-state functional connectivity fMRI, two studies also found an increased involvement of the right DLPFC within widespread functional brain networks in AVH+ patients^1,11^. However, one study reported a reduced functional connectivity between the right DLPFC and inferior frontal gyrus in AVH+ patients as compared to healthy controls^7^. Finally, current results could appear in contradiction with results from Ćurčić-Blake *et al*.^12^ showing higher Glx levels in the left DLFPC in AVH+ patients than in no-AVH patients since numerous studies have reported a functional coupling between NAA and Glx in healthy subjects^18^. However, a decoupling between NAA and Glx levels was observed in the DLPFC in patients with schizophrenia^19^. In the current study, we also found a positive correlation between NAA levels in the right DLPFC and the severity of AVH, reinforcing the link between the right DLPFC and AVH.

It is important to note that the findings of a significant difference in NAA levels between left and right DLPFCs in AVH+ patients but not in no-AVH patients are difficult to interpret in the absence of a group of healthy subjects. Interestingly, Brambilla *et al*.^20^ observed no difference in NAA levels between the right and left DLPFCs in healthy subjects, suggesting that NAA levels in the DLPFC showed no hemispheric lateralization in healthy subjects. Taken together, these findings suggested that a prefrontal imbalance in NAA levels might be linked to AVH presence. However, further studies including a group of healthy subjects are needed to test this imbalance hypothesis.

Finally, our findings highlight the role of the DLPFC in AVH and are in accordance with cognitive models of AVH. In these models, the DLPFC is described as a key structure involved in “top-down” and monitoring processes suggesting that AVH generation results from a failure in speech monitoring^21^. More specifically, cognitive models suggest that AVH generation results from a functional breakdown in appropriate monitoring of inner speech generation leading to misattribution of inner speech to externally perceived event. This misattribution may be supported by an abnormal “corollary discharge”, a mechanism by which the frontal regions can modulate the temporal area activity by sending inhibitory information^22^.

Some limitations may have influenced our results. First, our sample size was limited and only male patients were included. Although NAA levels in the prefrontal region seem not to be influenced by gender^23^, these results need to be confirmed by further studies including larger sample sizes and including female patients. Second, it has been reported that antipsychotic medication may modulate metabolic levels measured by MRS. Some studies reported that antipsychotic medication increased NAA levels in the prefrontal cortex^24^ and others reported that some antipsychotic such as risperidone can decrease NAA in the medial prefrontal cortex^25^. This discrepancy can be explained by clinical heterogeneity of patients with schizophrenia included in studies, level of treatment-resistance or by the diversity of antipsychotic treatments used in studies. Moreover, NAA alterations were also found in drug free subjects at-risk for developing schizophrenia^23^. In our study, AVH+ and no-AVH patients were all under pharmacological treatments and experienced treatment-resistant symptoms. There was no difference between groups regarding antipsychotic treatment in term of dosage (expressed as equivalent chlorpromazine per day), molecule (first and second generation of antipsychotic) and duration of illness. Thus, the between-group difference in NAA levels might not be related to a between-group difference in antipsychotic medication.

Third, methodological issues need to be considered. The strength of the magnetic field used in our study (1.5 T) can be considered as a limitation. However, NAA is one of the most abundant amino acid in the brain and 1.5 T is sufficient to quantify NAA levels with accuracy, as has been done in other studies^26^, even recently^27^. Similar NAA abnormalities in patients with schizophrenia were described by studies using a 1.5 T scan as well as by studies using higher magnetic field strength^26^. Moreover, although creatine has been commonly used as an internal reference to quantify metabolites levels in 1H-MRS studies, in this study we chose not to express NAA levels as a ratio to creatine. Indeed, some recent data suggested that the use of creatine as an internal reference may not be appropriate for psychiatric research^28^ since creatine levels were decreased in patients with schizophrenia compared to healthy subjects^28,29^ especially in the left but not right DLPFC^30^. Finally, the interpretation of NAA levels remains difficult to date. Whereas it is accepted that NAA can be used as a marker of mitochondrial activity and osmotic balance^14^, NAA levels can also reflect neuronal activity and energy metabolism^13,14^, leading some authors to conclude that NAA function remains unknown^31^.

In summary, the current study highlighted a relationship between AVH severity and DLPFC NAA levels in patients with schizophrenia. Whereas no-AVH patients showed no difference in NAA levels between left and right DLPFC, AVH+ patients showed higher NAA levels in the right DLPFC as compared to the left. Moreover, our findings suggested a close relationship between NAA levels in the right DLPFC and the severity of AVH in patients with schizophrenia. The hemispherical imbalance between DLPFCs should thus be taken into account in studies investigating pathophysiology of schizophrenic symptoms.

## Methods

The study was approved by a local ethics committee (CPP Sud Est 6, France) and all patients gave their written informed consent. All experiments were performed in accordance with relevant guidelines and regulations. Patients were referred to our research unit and have participated in clinical studies registered on clinicaltrials.gov database (NCT00870909 and NCT00875498). The datasets generated during and/or analyzed during the current study are available from the corresponding author on reasonable request.

### Participants

Twenty-seven male patients who met DSM IV-TR criteria for schizophrenia were enrolled in this study between February 2009 and January 2016. The severity of symptoms was assessed using the Positive and Negative Syndrome Scale (PANSS). The severity of AVH was assessed by the PANSS P3 item (i.e., hallucinations). Since all the AVH+ patients experienced mainly AVH, the P3 item was used to measure exclusively AVH severity in our study. Patients were divided into two subgroups based on the presence of daily treatment-resistant AVH (PANSS P3 item > 3, AVH+ patients) or the absence of AVH (PANSS P3 item ≤ 3, no-AVH patients). The cut off P3 ≤ 3 has been fixed according to remission definition proposed by Andreasen *et al*. (2005)^32^. The subgroup of AVH+ patients consisted of 15 patients and the subgroup of no-AVH patients consisted of 12 patients. Patients presented with treatment-resistant symptoms defined as the persistence of symptoms despite two well conducted antipsychotic treatments from different pharmacological classes, in sufficient dose and duration^33^. All included patients were treated with antipsychotic medication for at least three months without change in dose and still presenting with disabling treatment-resistant symptoms.

At inclusion, patients were treated with several antipsychotic agents from different class. In the AVH+ group, 3 patients received first generation antipsychotic, 8 patients received second-generation antipsychotic and 4 received a combination of both. In the no-AVH group, 1 patient received first generation antipsychotic, 10 patients received second-generation antipsychotic and 1 received a combination of both. No difference between groups was observed regarding the class of antipsychotic received (Fisher Exact Probability Test = 0.37).

### Image acquisition

**Figure 3:**
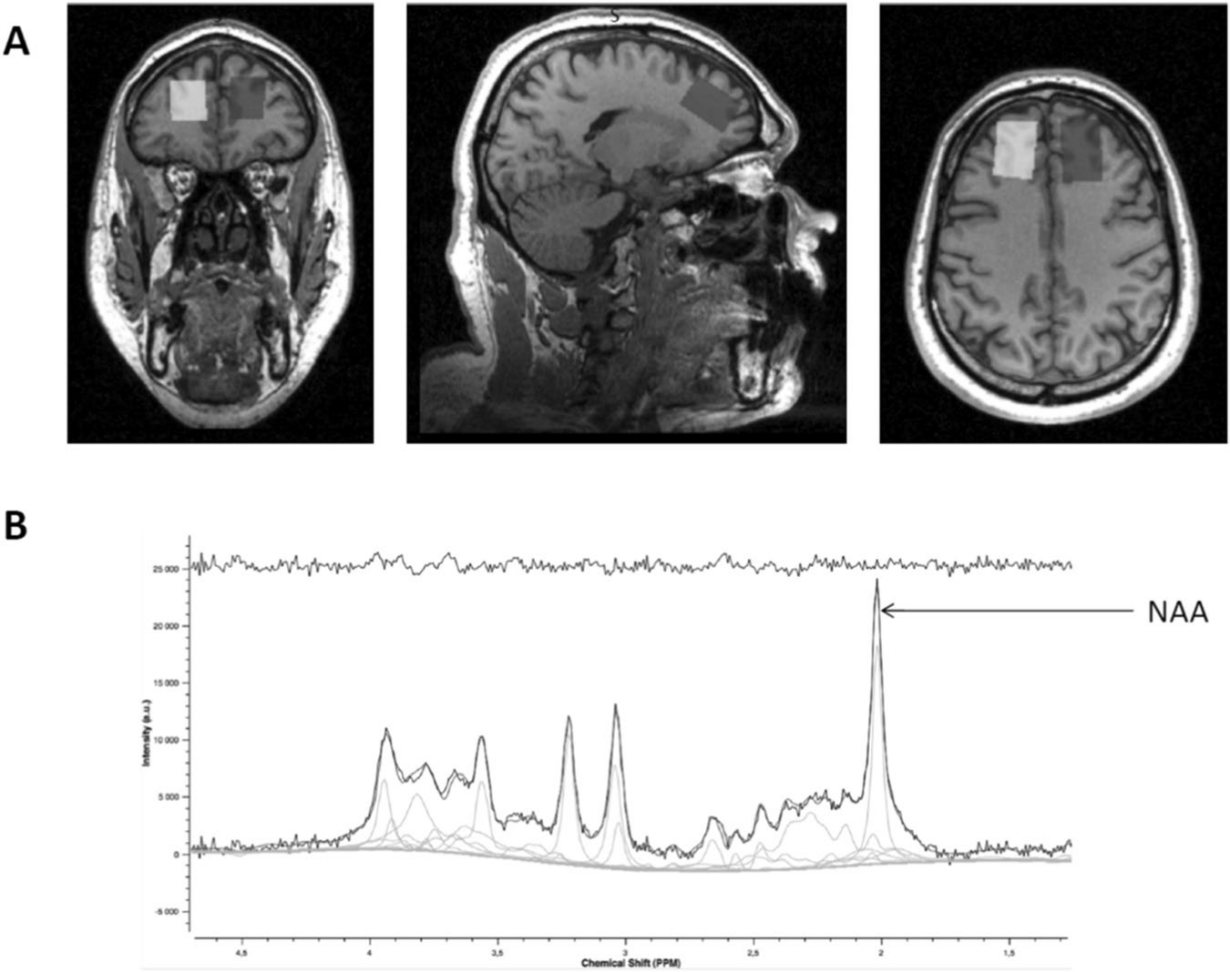
(**A**) Example of voxel placement on subject’s 3D anatomical MRI and (**B**) example of MRS spectra obtained with Tarquin 4.3.8 software.

Images were acquired on a 1.5-T Siemens Magnetom Sonata Maestro Class system equipped with a standard headcoil. A structural 3D image was obtained with a standard T1-weighted pulse sequence: 176 transverse slides, TR = 1970 ms, TE = 3.93 ms, flip angle of 15°, FOV = 256 × 256 mm, voxel size: 1.0 × 1.0 × 1.0 mm. *In vivo* ^1^H MRS measurements were acquired with PRESS sequence (TR = 1500 ms; TE = 30 ms; Spectral Bandwidth = 1000 Hz; Averages = 16; 1024 samples) with and without suppression of water signal (CHESS sequence). A 2 × 2 × 3 voxel of interest (12 cm^3^) was positioned on the 3D anatomical MRI to acquire signal in the left and the right DLPFC (Fig. 3).

### Data analysis

*In vivo* ^1^H MRS analyses were processed using Tarquin 4.3.8 software. Total NAA absolute concentration was determined and corresponded to the sum of NAA and N-Acetyl-Aspartyl-Glutamate (NAAG). NAA levels were calculated relative to the unsuppressed water signal from the same voxel.

Quantification reliability of results was assessed using Cramer-Rao lower bound (CRLB), a common metric for the goodness of spectral fit. The threshold for CRLB standard deviation values was set at 20%. Thus, a CRLB standard deviation value greater than or equal to 20% was considered unreliable.

Data quality was verified using the mean and standard deviation for CRLB, Signal to Noise Ratio (SNR), and Full width at half maximum (FWHM) and water peak in each group. No significant difference between groups was observed regarding CRLB, SNR, FWHM and water peak in the both DLPFCs (please see Supplementary Material 1).

Tissue segmentation was performed in both ROIs. No significant difference between groups was observed regarding CSF, gray matter and white matter composition per ROI in the both DLPFCs (please see Supplementary Material 2).

### Statistical analyses

Statistical analyses were performed with SPSS-22 (Statistical Package for the Social Sciences, version 22). Statistical significance was set at p < 0.05, two-tailed for all analyses. Sociodemographic and clinical characteristics of patients were compared using 2-tailed student t-tests for continuous variables and Fischer’s exact test for gender and antipsychotic medication class. MRS data quality and tissue segmentation were compared between groups (AVH+ and no-AVH) using 2-tailed student t-tests. Bonferroni correction for multiple comparisons was applied.

To investigate difference in NAA levels in the DLPFC between groups, NAA levels were entered in a mixed model ANOVA with group (AVH+, no-AVH) as between factor and laterality (left DLPFC, right DLPFC) as within factors. In case of significance, post hoc tests were performed with Bonferroni correction for multiple comparisons.

Relationships between AVH severity (measured by P3 item PANSS score) and NAA levels in the left and right DLPFC were investigated using Pearson correlation tests.

## Electronic supplementary material

Supplementary Material

## References

[1] Wolf ND (2011). Dysconnectivity of multiple resting-state networks in patients with schizophrenia who have persistent auditory verbal hallucinations. J. Psychiatry Neurosci..

[2] Jardri R, Pouchet A, Pins D, Thomas P (2011). Cortical activations during auditory verbal hallucinations in schizophrenia: a coordinate-based meta-analysis. Am. J. Psychiatry..

[3] Psomiades M (2016). Integrity of the arcuate fasciculus in patients with schizophrenia with auditory verbal hallucinations: A DTI-tractography study. Neuroimage Clin..

[4] Geoffroy PA (2014). The Arcuate Fasciculus in auditory-verbal hallucinations: a meta-analysis of diffusion-tensor-imaging studies. Schizophr. Res..

[5] Lawrie SM (2002). Reduced frontotemporal functional connectivity in schizophrenia associated with auditory hallucinations. Biol. Psychiatry..

[6] Steinmann S, Leicht G, Mulert C (2014). Interhemispheric auditory connectivity: structure and function related to auditory verbal hallucinations. Front. Hum. Neurosci..

[7] Sommer IE, Clos M, Meijering AL, Diederen KM, Eickhoff SB (2012). Resting state functional connectivity in patients with chronic hallucinations. PLoS One..

[8] Cui LB (2016). Putamen-related regional and network functional deficits in first-episode schizophrenia with auditory verbal hallucinations. Schizophr. Res..

[9] Clos M, Diederen KM, Meijering AL, Sommer IE, Eickhoff SB (2014). Aberrant connectivity of areas for decoding degraded speech in patients with auditory verbal hallucinations. Brain Struct. Funct..

[10] Mondino M, Brunelin J, Saoud M (2013). N-Acetyl-Aspartate Level is decreased in the prefrontal cortex in subjects at-risk for schizophrenia. Front. Psychiatry..

[11] Cui LB (2017). Disturbed Brain Activity in Resting-State Networks of Patients with First-Episode Schizophrenia with Auditory Verbal Hallucinations: A Cross-sectional Functional MR Imaging Study. Radiology..

[12] Ćurčić-Blake B (2017). Glutamate in dorsolateral prefrontal cortex and auditory verbal hallucinations in patients with schizophrenia: A 1H MRS study. Prog. Neuropsychopharmacol. Biol. Psychiatry..

[13] Moffett JR, Arun P, Ariyannur PS, Namboodiri AM (2013). N-Acetylaspartate reductions in brain injury: impact on post-injury neuroenergetics, lipid synthesis, and protein acetylation. Front. Neuroenergetics..

[14] Baslow MH, Burlina AP (2012). N-Acetylaspartate Metabolism Underlays the Structural and Functional Units of the Vertebrate Brain: A Bioenergetic Rationale for Clinical Observations of Changes in the Neuronal Biomarker “NAA” in many Human Brain Disorders. Bioenerg. Open Access..

[15] Kraguljac NV (2012). Neurometabolites in schizophrenia and bipolar disorder - a systematic review and meta-analysis. Psychiatry Res..

[16] Callicott JH (2000). Selective relationship between prefrontal N-acetylaspartate measures and negative symptoms in schizophrenia. Am. J. Psychiatry..

[17] Sigmundsson T (2003). Frontal lobe N-acetylaspartate correlates with psychopathology in schizophrenia: a proton magnetic resonance spectroscopy study. Schizophr. Res..

[18] Kraguljac NV, Reid MA, White DM, den Hollander J, Lahti AC (2012). Regional Decoupling of N-acetyl-aspartate and Glutamate in Schizophrenia. Neuropsychopharmacol..

[19] Coughlin JM (2015). Decoupling of N-acetyl-aspartate and glutamate within the dorsolateral prefrontal cortex in schizophrenia. Curr. Mol. Med..

[20] Brambilla P (2004). 1H MRS Study of Dorsolateral Prefrontal Cortex in Healthy Individuals before and after Lithium Administration. Neuropsychopharmacology..

[21] Allen P, Aleman A, McGuire PK (2007). Inner speech models of auditory verbal hallucinations: evidence from behavioural and neuroimaging studies. Int. Rev. Psychiatry..

[22] Ford JM, Mathalon DH (2005). Corollary discharge dysfunction in schizophrenia: Can it explain auditory hallucinations?. Int. J. Psychophysiol..

[23] Mondino M, Brunelin J, Saoud M (2013). N-Acetyl-Aspartate Level is decreased in the prefrontal cortex in subjects at-risk for schizophrenia. Front. Psychiatry..

[24] Szulc A (2013). Proton magnetic resonance spectroscopy changes after antipsychotic treatment. Curr. Med. Chem..

[25] Zong X (2015). N-acetylaspartate reduction in the medial prefrontal cortex following 8 weeks of risperidone treatment in first-episode drug-naïve schizophrenia patients. Sci. Rep..

[26] Steen RG, Hamer RM, Lieberman JA (2005). Measurement of brain metabolites by 1H magnetic resonance spectroscopy in patients with schizophrenia: a systematic review and meta-analysis. Neuropsychopharmacol..

[27] Birur B, Kraguljac NV, Shelton RC, Lahti AC (2017). Brain structure, function, and neurochemistry in schizophrenia and bipolar disorder-a systematic review of the magnetic resonance neuroimaging literature. NPJ Schizophr..

[28] Ongür D, Prescot AP, Jensen JE, Cohen BM, Renshaw PF (2009). Creatine abnormalities in schizophrenia and bipolar disorder. Psychiatry Res..

[29] Wijtenburg SA, Yang S, Fischer BA, Rowland LM (2015). *In vivo* assessment of neurotransmitters and modulators with magnetic resonance spectroscopy: application to schizophrenia. Neurosci. Biobehav. Rev..

[30] Kalayci D, Ozdel O, Sozeri-Varma G, Kiroglu Y, Tumkaya S (2012). A proton magnetic resonance spectroscopy study in schizoaffective disorder: comparison of bipolar disorder and schizophrenia. Prog. Neuropsychopharmacol. Biol. Psychiatry..

[31] Bhakoo, K.K. N-acetyl-aspartate (NAA) metabolism in *Advances in Neurobiology* (ed. Lathja, A.) 1075-1093 (Springer, New York, 2012).

[32] Andreasen NC (2005). Remission in schizophrenia: proposed criteria and rationale for consensus. Am. J. Psychiatry..

[33] Kane JM, Honigfeld G, Singer J, Meltzer H (1988). Clozapine in treatment-resistant schizophrenics. Psychopharmacol. Bull..

