# Supplementary material for: N-Acetyl-Aspartate in the dorsolateral prefrontal cortex in men with schizophrenia and auditory verbal hallucinations: A 1.5 T Magnetic Resonance Spectroscopy Study
Source: Sci Rep. 2018 Mar 7;8:4133. doi: 10.1038/s41598-018-22597-y (PMC5841306; doi:10.1038/s41598-018-22597-y)
Supplement: Supplementary file 1 — Supplementary Material [file 41598_2018_22597_MOESM1_ESM.docx]

**N-Acetyl-Aspartate in the dorsolateral prefrontal cortex in men with schizophrenia and auditory verbal hallucinations: A 1.5T Magnetic Resonance Spectroscopy Study.**

Marion PSOMIADES, Marine MONDINO, Clara FONTENEAU, Rémy BATION, Frederic HAESEBAERT, Marie-Françoise SUAUD-CHAGNY, Jérome BRUNELIN.

**Supplementary Material 1. MRS data quality**

|  | AVH+ | no-AVH | P |
| --- | --- | --- | --- |
| CRLB NAA  Right DLPFC  Left DLPFC | 4.5 (1.4)  7.9 (3.5) | 6.6 (2.8)  6.5 (4.2) | 0.14  1 |
| SNR |  |  |  |
| Right DLPFC | 26.4 (5.7) | 25.2 (6.3) | 1 |
| Left DLPFC | 24.6 (4.6) | 27.1 (4.7) | 1 |
| FWHM NAA (ppm) |  |  |  |
| Right DLPFC | 0.053 (0.013) | 0.065 (0.020) | 0.79 |
| Left DLPFC | 0.068 (0.015) | 0.061 (0.016) | 1 |
| Water width (ppm)  Right DLPFC  Left DLPFC | 0.087 (0.007)  0.105 (0.015) | 0.095 (0.013)  0.092 (0.015) | 0.49  0.36 |

Data quality was verified using the mean and standard deviation for CRLB, Signal to Noise Ratio (SNR), and Full width at half maximum (FWHM) and water peak in each group. No significant difference between groups was observed regarding CRLB, SNR, FWHM and water peak in the both DLPFCs.

MRS data quality was compared between groups (AVH+ and no-AVH) using 2-tailed student t-tests. Bonferroni correction for multiple comparisons was applied.

**Supplementary Material 2. Tissue segmentation**

|  | AVH+ | no-AVH | P |
| --- | --- | --- | --- |
| Grey matter (%)  Right DLPFC  Left DLPFC | 41.7 (3.4)  39.4 (4.3) | 37.6 (6.6)  36 (6.6) | 0.30  0.73 |
| White matter (%) |  |  |  |
| Right DLPFC | 52.2 (4.6) | 53.7 (9.2) | 1 |
| Left DLPFC | 54.1 (5.0) | 55.1 (9.5) | 1 |
| CSF (%) |  |  |  |
| Right DLPFC | 5.9 (3.7) | 8.5 (5.3) | 0.87 |
| Left DLPFC | 6.3 (3.6) | 8.8 (4.8) | 0.84 |

Tissue segmentation was performed in both ROIs (right and left DLPFC) in patients with AVH and in patients with no AVH.

No significant difference between groups was observed regarding CSF, gray matter and white matter composition per ROI in the both DLPFCs.

Tissue segmentation was compared between groups (AVH+ and no-AVH) using 2-tailed student t-tests. Bonferroni correction for multiple comparisons was applied.
